# Decreased nerve conduction velocity may be a predictor of fingertip dexterity and subjective complaints

**DOI:** 10.1007/s00221-023-06556-2

**Published:** 2023-01-20

**Authors:** Yuki Fukumoto, Takuya Wakisaka, Koichi Misawa, Masanobu Hibi, Toshiaki Suzuki

**Affiliations:** 1 Graduate School of Health Sciences, Graduate School of Kansai University of Health Sciences, 2-11-1 Wakaba, Sennan-gun, Kumatori, Osaka, 590-0482 Japan; 2 Biological Science Laboratories, Kao Corporation, 2-1-3 Bunka, Sumida-ku, Tokyo, 131-8501 Japan

**Keywords:** Age-related changes, Fingertip dexterity, Subjective complaints, Motor nerve conduction velocity, Sensory nerve conduction velocity

## Abstract

We examined the causes of decreased fingertip dexterity in elderly individuals with an aim to improve their quality of life by improving their activities of daily living. We calculated nerve conduction velocity, absolute error during force adjustment tasks, and fingertip dexterity test scores for 30 young (21–34 years old) and 30 elderly (60–74 years old) participants to identify age-related changes. We also assessed subjective complaints of pain, motor function, and numbness. Motor nerve (young: 55.8 ± 3.7 m/s; elderly: 52.2 ± 5.0 m/s) and sensory nerve (young: 59.4 ± 3.4 m/s; elderly: 55.5 ± 5.3 m/s) conduction velocities decreased in an age-dependent manner. Moreover, the decrease of motor nerve conduction velocity was associated with decreased fingertip dexterity (objective index), while the decrease of sensory nerve conduction velocity was associated with subjective complaints of pain and motor function (subjective index).

## Introduction

The activities of daily living (ADLs) are important factors when considering the health status of elderly individuals. The ADLs consist of basic activities, e.g., moving and maintaining posture, and personal activities, e.g., changing clothes and eating. The ADLs correlate with the quality of life (QOL) (Ueya and Koyama 2011), and as individuals age, their ability to perform the ADLs declines, which may result in a decrease in QOL. In particular, it is highly desirable to maintain the ADLs in elderly individuals so they can achieve a high QOL in the long term. Many ADLs require the ability to perform accurate pinching movements, e.g., putting on and taking off clothing. Therefore, elderly individuals frequently complain of subjective symptoms related to fingertip dexterity. However, there are also many cases in whom subjective symptoms are reported, but there are no objective findings on clinical examination and no causative disease is found, so-called “indefinite complaints.” Even in the absence of a medical diagnosis, poor fingertip dexterity significantly reduces the ADLs and QOL (Canning et al. 2000; Duque et al. 1995), and therapists should appreciate the relationship between fingertip dexterity and daily life and aim to resolve the associated problems (Gonzalez et al. 2015; Metcalf et al. 2008). Accordingly, we thought that if the causes of decreased fingertip dexterity, which can be regarded as a complaint of the elderly, can be elucidated and applied to rehabilitation, it would contribute to an improvement of QOL by improving the ADLs.

It is generally accepted that fingertip dexterity declines with age (Bennett and Castiello 1994; Michimata et al. 2008). Marmon et al. (2011) used a force regulation task to examine pinch movements and a pegboard to assess fingertip dexterity in three groups: young adults aged 18–36 years, middle-aged adults aged 40–60 years, and elderly adults aged ≥ 65 years. They found that fingertip dexterity and the maximal voluntary contraction (MVC) strength of pinch force decrease in an age-related manner. Furthermore, muscle weakness causes a reduction in fingertip dexterity. Even in the absence of muscle atrophy, neurological factors can induce muscle weakness through reduced muscle output. The reduction in muscle strength associated with decreased muscle output increases the unsteadiness of muscle output during movement (Enoka et al. 2003; Hamilton and Wolpert 2002). In other words, reduced muscle output increases the variability in the accuracy of the trajectory and final position of motor movements, and we consider that reduced muscle output is a cause of reduced finger dexterity. In this way, organic and neurological factors may contribute to the decline in muscle strength in the elderly. First, organic factors may be caused by a decrease in the strength and mass of skeletal muscle, which is a common explanation (Brown et al. 1995; Carmeli et al. 2003; Enoka et al. 2003; Grabiner and Enoka 1995). Second, neurological factors include reduced nerve conduction velocity (Dorfman and Bosley 1979; Kurokawa et al. 1999; Mackenzie and Phillips 1981), changes in motor units (Lexell 1997), and impaired transmission (Tintignac et al. 2015).

Motor nerve conduction velocity (MCV) indirectly reflects how motor units transmit action potentials and may be a factor explaining muscle weakness. We hypothesized that a decrease of MCV may induce a loss of synchrony between the activity of multiple muscles and/or an error between the actual timing of muscle activity and the timing predicted by the internal model. The insufficient activation of motor units, i.e., inadequate rate at which motor units generate action potentials, is generally observed with aging, and this may contribute to the age-related decline in muscle strength and power (Hunter et al. 2016). Therefore, we thought that minor nerve dysfunction due to decreased MCV could trigger motor dysfunction. Thus, motor nerve function could be one factor related to fingertip dexterity and muscle strength.

However, we use sensory information to move our bodies (Sheridan 1984), and information obtained from sensory nerves is important. Therefore, sensory nerve impairment affects the feedback control of movement, which in turn has a negative impact on motor performance (Kelso 1982). Afferent information from peripheral sensors is combined with predictive signals generated by an internal motor model (Wolpert et al. 1995). The brain uses the forward model to predict the sensory consequences of its actions, making the system robust to the delays and noise associated with sensory processing (Wolpert et al. 2011). Performance is stabilized by continuously comparing sensory information from the environment with the predicted movement outcome. Therefore, changes in the accuracy of sensory information relative to predicted movement outcomes will have widespread effects on motor control (Edwards et al. 2012). For example, the sensory consequences of spontaneous actions are perceived weakly compared to the same externally generated sensory events (Shergill et al. 2003). Thus, the attenuation of sensory information irrelevant to motor execution facilitates the capacity to optimize voluntary movements (Brown et al. 2013). However, normal aging results in reduced sensory sensitivity and increased sensory noise in peripheral and central processing (Konczak et al. 2012). Given the pronounced sensory and motor changes associated with aging, somatosensory inhibition is expected to have functional effects. Increased somatosensory noise (Decorps et al. 2014) and increased motor variability (Contreras-Vidal et al. 1998) amplify the weighting of sensory-motor predictions and contribute to the greater attenuation of sensory behavioral outcomes. Thus, we accept a certain consensus that aging decreases somatosensory sensitivity, especially during self-generated movement (Klever et al. 2019; Wolpe et al. 2016). As the reported impairment of somatosensory signals is reflected in the intensity of sensory input, it is necessary to identify age-related modulation of the underlying sensory afferents. Therefore, when there are subjective symptoms, but no objective findings, on clinical examination and no causative disease is found, it is possible that there is a sensory nerve abnormality, even if there is no motor nerve problem or that a comprehensive examination of motor and sensory nerves may help to determine the cause of the symptoms. We hypothesized that subjective complaints are related to sensory nerve abnormalities and that decreased fingertip dexterity is related to motor nerve abnormalities. Furthermore, we recognized the need for a comprehensive study of motor and sensory nerves together with decreased fingertip dexterity.

While the age-related loss of fingertip dexterity is problematic, the neurological root cause has yet to be resolved fully. There have been no reports of comprehensive solutions from both motor and sensory perspectives, and the relationship between motor skills and neurological factors remains unclear. Therefore, assessing subjective complaints in ADLs and how they relate to physiological changes is necessary. In order to explain the decline in fingertip dexterity in the elderly in terms of neurological factors, it is important to know what is modulated by aging, compared to younger individuals who generally do not exhibit neurological factors and fingertip dexterity problems. In other words, neurological factors that show changes when comparing younger and elderly individuals may be linked to the cause of reduced fingertip dexterity in the elderly. In the present study, we aimed to clarify the relationship between subjective symptoms and reduced fingertip dexterity from the perspective of the motor and sensory nervous systems, and to examine if there is a causal relationship between fingertip dexterity and neurological factors by using structural equation modeling.

## Materials and methods

### Participants

Power analysis was conducted using G*power with the following factors: “a priori: compute required sample size given α, power, and effect size,” “difference between two independent means (two groups), difference between independent mean (two groups) or Wilcoxon Mann–Whitney test (two groups),” effect size = 0.8, alpha error probability = 0.05, and power (1-β error probability) = 0.8. As a result, the required sample size was calculated to be 52–54; therefore, we included 60 participants in this study, allowing for the possibility of some participants dropping out. The participants consisted of 30 healthy young adults (16 males and 14 females, 21–34 years old, mean age 24.8 ± 4.3 years) and 30 healthy elderly adults (15 males and 15 females, 60–74 years old, mean age 69.2 ± 3.5 years) living in Kumatori Town, Japan. All participants self-reported that they were right-handed.

Participants were excluded from the study if they had a medical diagnosis of disease or carpal tunnel syndrome. The medical diagnosis of disease was based on interviews with the participants, and no additional judgment by a physician was required because of the nature of the complaints and to take these factors into account on the assumption that a potential neurological problem can exist even in the absence of a clear medical diagnosis. All participants provided informed consent prior to the commencement of the study. The experiments were conducted in accordance with the Declaration of Helsinki. This study was conducted with the approval of the Research Ethics Review Committee of Kansai University of Health Science (approval number: 21–09) and the Human Ethics Committee of Kao Corporation (approval number: S264-191,113).

### Study procedures

Three processes were conducted in sequence: evaluation of neurological function; evaluation of fingertip dexterity; and evaluation of the degree of sensory impairment. All measurements were performed in a laboratory with the temperature controlled at 25 ℃. In addition, skin temperature was confirmed to be 35 °C or higher.

### Evaluation of neurological function

In this study, MCV, sensory nerve conduction velocity (SCV), and sympathetic nerve activity were recorded to evaluate neurological function.

### MCV recording conditions

**Fig. 1:**
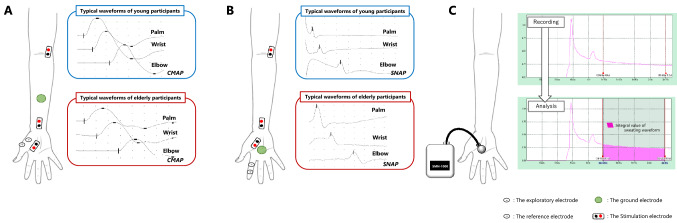
Evaluation of neurological function. **A** Electrode attachment sites for the motor nerve conduction test. **B** Electrode attachment sites for the sensory nerve conduction test. **C** Measurement of sweating volume from the palmar region and the obtained sweating waveforms. Nerve conduction velocity was recorded first, followed by measurement of sympathetic nerve activity. CMAP, compound motor action potential; SNAP, sensory nerve action potential

Viking Quest ver. 9.0 (Natus Medical, Inc.) was used with a band frequency of 20 to 3 kHz and sampling frequency of 10 kHz. Under the recording conditions, stimulation intensity was set to maximal suprastimulus. The stimulation sites were the palm (5 cm distal to the palmar transverse folds of the carpus), wrist joint (3 cm proximal to the palmar transverse folds of the carpus), and elbow joint (transverse fold of the elbow fossa along the brachial artery), and the median nerve was electrically stimulated from each stimulation point. For the motor nerves, the optimal stimulation site where the compound motor active potential showed the maximum amplitude was identified by repeated moderate stimulation, and stimulation intensity was further increased at that site to determine the intensity at which the amplitude of the compound motor active potentials was maximized. These processes excited all components of the targeted nerve fibers, resulting in consistent compound motor active potentials. All participants were given consistent one maximal suprastimulus (10–20 mA) at each stimulus point. One maximal supramaximal stimulus at each stimulus point produced one corresponding waveform (compound motor action potential). The exploratory electrode for recording muscle action potentials from the muscle belly was placed on the abductor pollicis brevis, and the reference electrode to record muscle action potentials from above the bone (almost zero) was placed on the dorsal side of the right first metacarpal head. The ground electrode, which served as a common return for the current circuit, was situated in the middle of the right forearm (Kimura 2013) (Fig. 1A). To measure nerve conduction velocity accurately, the distance between adjacent stimulation points should be at least several centimeters, ideally ≥ 10 cm (Kimura 2013). Since nerve conduction velocity in the forearm segment (elbow joint–wrist joint stimulation) satisfying this condition is related to force control (Chang et al. 2008), the same procedure was followed and MCV was calculated in the forearm segment. MCV (elbow-wrist) was separately calculated for each participant upon completion of electrical stimulation at all stimulation points and extraction of the corresponding waveforms. In other words, the MCV of each subject's forearm was calculated by dividing the forearm length between the elbow and wrist joint stimulation sites by the difference in latency of compound motor action potential appearance obtained from the elbow and wrist joint stimulation sites. In addition, terminal latency was recorded from palmar stimulation. Three components are involved in motor nerve terminal latency: conduction time from the stimulation site to the nerve terminal; time for transmission to the muscle endplate at the neuromuscular junction; and depolarization of the endplate followed by the evocation of action potentials at the muscle cell membrane.

### SCV recording conditions

For the sensory nerves, the antidromic method was used to confirm the negative waveform rise time of sensory nerve action potentials. Sensory nerve action potentials are very small and are generally difficult to distinguish from noise. Therefore, by performing an additive averaging process of 20 waveforms, the noise is canceled out and only the sensory nerve action potential waveform is revealed. Therefore, 20 stimuli were used to calculate sensory nerve action potentials, and one waveform at each stimulus point was extracted by performing an additive averaging of 20 waveforms. In other words, an additive averaging process was performed at the end of 20 stimuli at each stimulus point, and the one waveform (sensory nerve action potential) corresponding to the stimulus point was obtained. To prevent compound motor action potentials from being included in the sensory nerve action potential waveforms, the increase in stimulus intensity was interrupted before the appearance of compound motor action potentials. This was done with the intention of checking the exact sensory nerve action potential rise latency by eliminating contamination with compound motor action potentials. Furthermore, the latency of motor nerve stimulation includes the neuromuscular transmission time, but the latency of sensory nerves is the nerve conduction time itself from the nerve stimulation site to the recording electrode, so the conduction velocity can be calculated even with stimulation at a single site. However, SCV was determined by electrically stimulating the palm, wrist joint, and elbow joint because we wanted to calculate the velocity at the forearm segment (elbow joint–wrist joint stimulation) as in MCV, rather than from each stimulation point. Therefore, SCV (elbow-wrist) was separately calculated for each participant upon completion of electrical stimulation at all stimulation points and extraction of the corresponding waveforms. In other words, the SCV of each subject's forearm was calculated by dividing the forearm length between the elbow and wrist joint stimulation sites by the difference in latency of sensory nerve action potential (After performing an additive averaging of 20 waveforms each stimulation sites) appearance obtained from the elbow and wrist joint stimulation sites. Sensory nerve terminal latency from palmar stimulation is reflected by the conduction time from the stimulation site to the recording electrode. To derive sensory potentials along the nerve proximal and distal to the tip of the index finger, the exploratory electrode was located at the right proximal interphalangeal joint (index fingertip), the reference electrode was placed 3 cm distal to the exploratory electrode, and the ground electrode was attached to the center of the right palm (Kimura 2013) (Fig. 1B).

### Sympathetic nerve activity recording conditions

Fingertip dexterity, which is evaluated by motor function, is transiently decreased by mental strain (Furuya et al. 2021). Temporary loss of ability due to mental strain is influenced by the experimental environment and is unlikely to reflect a true loss of fingertip dexterity that interferes with daily life. Therefore, we investigated the relationship between the degree of true fingertip dexterity loss and nerve conduction velocity, which exists independently of the environment, and the temporary factors caused by mental tension should be excluded. After recording nerve conduction velocity, sympathetic nerve activity was analyzed through the amount of sweating in the palms to evaluate psychogenic strain (Takahashi et al. 2017; Zheng et al. 2015). An SMN-1000 (SKINOS, Inc.) ventilated capsule sweat meter with flow compensation was used for measurement, and the average value per 1 min was obtained by dividing the integral value of the sweating waveform by the measurement time and used as an index of sympathetic nerve activity (Fig. 1C).

### Evaluation of fingertip dexterity

**Fig. 2:**
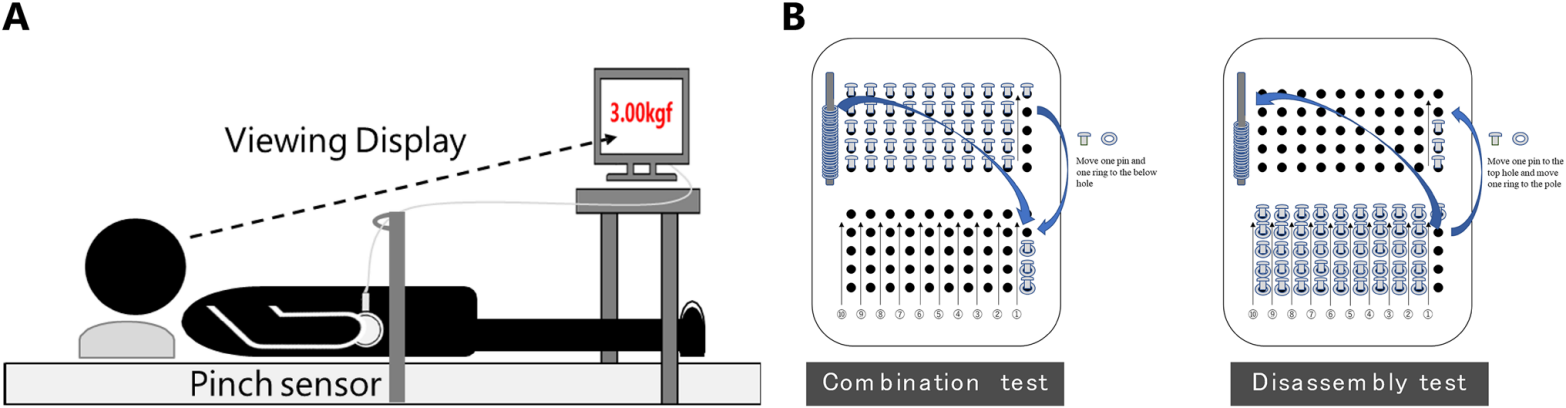
Evaluation of fingertip dexterity. **A** Measurement of pinch force using a pinch sensor. The waveform was analyzed during adjustment to the specified value and the error was calculated by the absolute value. **B** A pegboard used to calculate the fingertip dexterity test scores. The number of round rivets and washers that could be combined or disassembled within a certain time was evaluated

The index of fingertip dexterity was based on errors during the force adjustment task and fingertip dexterity test scores using a pegboard (Marmon et al. 2011). In the force adjustment task, the participant was in a supine position and able to move their upper limbs freely. However, the pinch sensor was suspended above the participant's palm and could be pinched easily, so the participant did not need to move their upper limbs. The force adjustment task was performed with the thumb and tip of the index finger of the dominant hand. The back of the pinch sensor was secured to the belly of the index finger, and pinch force was exerted by pushing with the belly of the thumb. First, the maximum value during a 10 s period in which the participant exerted maximum effort was taken as 100% MVC, from which 10% MVC was calculated. The error in exerting a 10% MVC pinch force for 10 s was used as an index of fingertip dexterity (Fukumoto et al. 2016, 2021) because elderly participants have difficulty in exerting a stable amount of force of approximately 10% MVC during isometric contractions (Griffin et al. 2009). Visual feedback was provided by the numeric value of pinch force (kgf) displayed on a monitor. Error calculation can be indicated by an absolute or relative value. Relative values can demonstrate whether the participant overshoots or undershoots the required force, but if overshoots and undershoots are mixed in a 10 s period, averaging them may cancel both out, leading to erroneous results. Therefore, a 10 s countdown was started when the subject reached the required pinch force, in which the absolute error was calculated by subtracting the exerted value from the specified value and converting it to an absolute value. Pinch force was AD converted at a sampling frequency of 1 kHz with Vital Recorder2 (KISSEI COMTEC, Inc.) electromyography recording software and analyzed using BIMUTAS-Video (KISSEI COMTEC, Inc.), a versatile bioanalysis system (Fig. 2A). The fingertip dexterity test consisted of a combination test and a disassembly test using a pegboard (T.K.K.1306 Hand-Finger Testing Device; Takei Scientific Instruments Co., Ltd.) (Nishiwaki et al. 2019) (Fig. 2B). When using the pegboard, the participant sat in a back-rested chair position, with the upper arms in a drooping, comfortable position, slightly flexed at the elbow joint, and the hands reaching near the pegboard on the desk. In the combination test, the participant removed a round rivet from a hole in the upper half of the pegboard with their right hand and simultaneously removed a washer from a rod with their left hand, fitted the washer to the round rivet, supported the round rivet with their right fingertip so that the washer did not fall off, and inserted the round rivet into the corresponding hole in the lower half of the pegboard. In the disassembly test, the participant pulled out a round rivet with a washer using their right hand from the lower half of the pegboard. At the same time, they used the tip of the left index finger to slide the washer from the round rivet. The participant then placed the washer on the washer rod and inserted the round rivet into the corresponding hole in the upper half of the pegboard. The combination test was performed for 90 s and the disassembly test was performed for 60 s as quickly as possible. The fingertip dexterity test score was calculated from the total scores of the combination and disassembly tests using the conversion table provided in the pegboard guide (The Employment Security Bureau of the Ministry of Health 2014) (Table 1).

**Table 1:** Conversion table

Combination test				Disassembly test			
Crude score	Conversion score	Crude score	Conversion score	Crude score	Conversion score	Crude score	Conversion score
50	156	10	5	50	91	10	− 9
49	152	9	2	49	89	9	− 11
48	148	8	− 2	48	86	8	− 14
47	144	7	− 5	47	84	7	− 16
46	140	6	− 9	46	81	6	− 19
45	136	5	− 12	45	79	5	− 21
44	132	4	− 16	44	76	4	− 24
43	128	3	− 19	43	74	3	− 26
42	124	2	− 23	42	71	2	− 29
41	120	1	− 26	41	69	1	− 31
40	116	0	− 30	40	66	0	− 34
39	112			39	64		
38	108			38	61		
37	104			37	59		
36	100			36	56		
35	96			35	54		
34	92			34	51		
33	88			33	49		
32	84			32	46		
31	80			31	44		
30	76			30	41		
29	72			29	39		
28	68			28	36		
27	65			27	34		
26	62			26	31		
25	59			25	29		
24	56			24	26		
23	52			23	24		
22	48			22	21		
21	44			21	19		
20	40			20	16		
19	37			19	14		
18	33			18	11		
17	30			17	9		
16	26			16	6		
15	23			15	4		
14	19			14	1		
13	16			13	− 1		
12	12			12	− 4		
11	9			11	− 6		

### Evaluation of the degree of sensory impairment

The participants were asked about subjective complaints of pain, motor function, and numbness using an original questionnaire developed from the Japanese version of the Patient-Rated Wrist Evaluation (Imaeda et al. 2010) and the Japanese version of the pain DETECT Questionnaire (Matsubayashi et al. 2013). The questionnaire used an 11-point Likert scale (0–10), with higher numbers indicating stronger complaints.

### Data analysis

Fingertip dexterity test scores that showed normality in the Shapiro–Wilk test were compared between the young and elderly groups using Student's *t* test. MCV, SCV, motor/sensory nerve terminal latency, sweating waveform integral, MVC, and absolute error that did not show normality in the Shapiro–Wilk test were compared between the young and elderly groups using the Mann–Whitney *U* test. Furthermore, the causal relationship leading to decreased fingertip dexterity in the elderly was examined by structural equation modeling using indices that were found to change with age. Structural equation modeling is a statistical model that extends factor analysis and multiple regression analysis to estimate the presumed causal relationships between latent and observed variables. Structural equation modeling also introduces latent variables that cannot be observed directly; therefore, it is possible to analyze the association between variables without a control group (Brown 2006; Browne and Cudeck 1993). In structural equation modeling, the standardized coefficients obtained allow us to quantify the influence of each factor. This makes it possible to evaluate the entire hypothesis logic that has been constructed, thus preventing repeated analyses and the accumulation of errors, and allows complex relationships between multiple data to be obtained numerically in a single analysis. Model fit was assessed by a goodness of fit index and root mean square error of approximation, and standardized estimates indicated the strength of the relationship between variables. For the degree of sensory impairment, the Mann–Whitney *U* test was used to compare pain, motor function, and numbness in the young and elderly groups, since they were assessed on an ordinal scale. The inclusion of data that do not follow normality or contain too many identical values (e.g., subjective evaluation) may bias the results of correlation analysis. Therefore, to address each concern, correlations with MCV and SCV were calculated for absolute error, fingertip test score, pain, motor function, and numbness using Spearman's rank correlation coefficient. The score of the fingertip dexterity test and the absolute error from 10% MVC were analyzed within each age group. However, in order to extract the abnormal characteristics of age-related modulation, the analysis should not include young subjects who generally do not exhibit neurological factors or fingertip dexterity problems, but should only use data from elderly subjects who have age-related modulation. Therefore, comparisons of the associations between MCV or SCV and each index were performed using only the data from the elderly group; bivariate comparisons were made separately, rather than multiple comparisons for which correlation matrices were created. The level of significance was set at less than 5%, and SPSS Statistics version 26.0 (IBM, Inc.) and Amos version 26.0 (IBM, Inc.) were used as statistical analysis software. Furthermore, for Student’s *t* test, the *t* value was used, and for the Mann–Whitney *U* test, the effect size (*r*) was calculated based on the *z* value.

## Results

### Neurological function

#### MCV

**Fig. 3:**
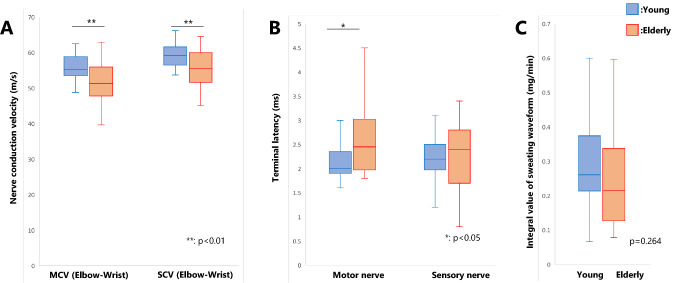
Neurological function. **A** Nerve conduction test results. Motor nerve conduction velocity and sensory nerve conduction velocity were both decreased in the elderly group. **B** Terminal latency. Only the motor nerves were delayed in the elderly group. **C** Sweating waveform integrals. There was no difference between both groups. MCV, motor nerve conduction velocity; SCV, sensory nerve conduction velocity

The median MCV (mean ± standard deviation [SD]) of the elbow-wrist for the young and elderly groups was 55.8 ± 3.7 and 52.2 ± 5.0 m/s, respectively, and the motor nerve latency (mean ± SD) of the young and elderly groups was 2.16 ± 0.43 and 2.57 ± 0.75 ms, respectively. MCV and motor nerve latency were decreased in the elderly group compared to the young group (MCV: *p* = 0.005, *r* = 0.51; motor nerve latency: *p* = 0.032, *r* = 0.39) (Fig. 3A, B).

#### SCV

The median SCV (mean ± SD) of the elbow–wrist for the young and elderly groups was 59.4 ± 3.4 and 55.5 ± 5.3 m/s, respectively, and the sensory nerve latency (mean ± SD) of the young and elderly groups was 2.19 ± 0.44 and 2.24 ± 0.70 ms, respectively. SCV was decreased in the elderly group compared to the young group (*p* = 0.005, *r* = 0.51). However, there was no difference in sensory nerve latency between both groups (*p* = 0.454, *r* = 0.14) (Fig. 3A, B).

### Sympathetic nerve activity

There was no difference in sweating waveform integrals between both groups (*p* = 0.264, *r* = 0.20) (Fig. 3C).

### Fingertip dexterity

**Fig. 4:**
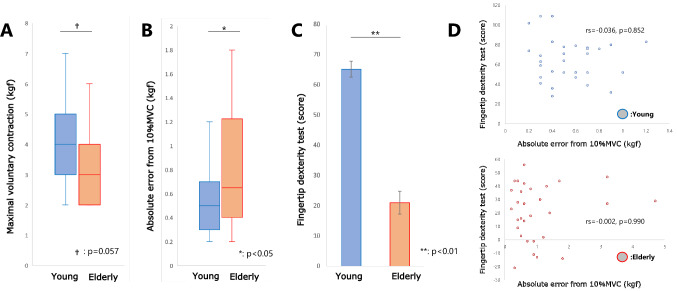
Fingertip dexterity. **A** Intensity of maximal voluntary contraction (MVC), which showed a decreasing trend in the elderly group. **B** Absolute error, which was increased in the elderly group. **C** Fingertip dexterity test score, which was lower in the elderly group. **D** Upper panel shows a scatter plot of the absolute error and fingertip dexterity test score, which revealed no correlation in each age group; lower panel shows the fingertip dexterity test score, which demonstrated no correlation in each age group

Compared to the young group, the elderly group tended to have weaker MVC strength (*p* = 0.057, *r* = 0.35), larger absolute error (*p* = 0.037, *r* = 0.38), and lower fingertip dexterity test scores (*p* < 0.001, *r* = 0.73). In terms of absolute error and fingertip dexterity test scores, the elderly group showed lower fingertip dexterity than the young group, but there was no correlation between the two indices (young: *rs* = − 0.036, *p* = 0.852; elderly: *rs* = − 0.002, *p* = 0.990) (Fig. 4).

### Degree of sensory impairment

The elderly group, which had no medically diagnosed conditions, had stronger subjective complaints of pain (*p* = 0.006, *r* = 0.50), motor function (*p* < 0.001, *r* = 0.73), and numbness (*p* = 0.003, *r* = 0.55) than the young group (Table 2).

**Table 2:** Subjective complaints

	Subjective pain complaints				Subjective functional complaints				Subjective numbness complaints			
	Score of young	Score of elderly	*P* value	effect size(r)	Score of young	Score of elderly	*P* value	Effect size(r)	Score of young	Score of elderly	*P* value	Effect size(r)
N1	0	5	0.006	0.50	0	0	< 0.001	0.73	0	3	0.003	0.55
N2	0	0			0	0			0	7		
N3	0	0			0	0			0	0		
N4	0	0			0	0			0	2		
N5	0	5			0	3			0	0		
N6	0	0			0	8			0	0		
N7	0	0			0	0			0	0		
N8	0	0			0	4			0	0		
N9	0	1			0	3			0	0		
N10	0	0			0	0			0	0		
N11	0	0			0	0			0	0		
N12	1	2			0	1			0	1		
N13	2	1			0	6			0	3		
N14	0	0			0	0			0	0		
N15	0	0			0	0			0	0		
N16	0	4			0	9			0	0		
N17	0	5			0	4			0	6		
N18	0	0			0	0			0	0		
N19	4	1			0	8			0	0		
N20	0	0			0	3			0	5		
N21	0	3			0	0			0	0		
N22	0	4			0	2			0	0		
N23	0	0			0	0			0	0		
N24	0	0			0	0			0	2		
N25	0	0			0	0			0	0		
N26	0	3			0	1			0	0		
N27	0	0			0	0			0	0		
N28	0	0			0	0			0	0		
N29	0	0			0	0			0	0		
N30	0	3			0	2			0	0		

### Relationship between each indicator

**Fig. 5:**
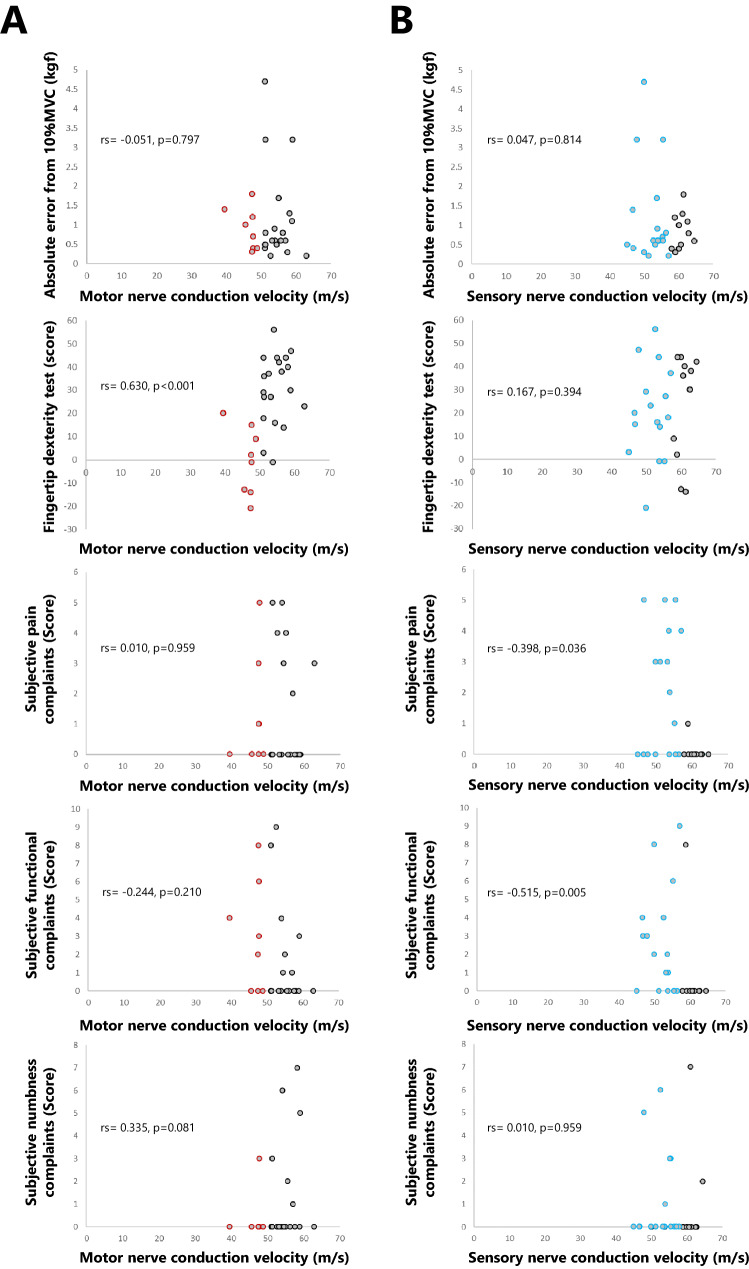
Relationship between nerve conduction velocity, fingertip dexterity, and subjective complaints. **A** Motor nerve conduction velocity (MCV). **B** Sensory nerve conduction velocity (SCV). Each scatter plot represents, from top to bottom, absolute error, fingertip dexterity test score, pain, function, and numbness; MCV was positively correlated with the fingertip dexterity test score, while SCV was negatively correlated with pain and function. Red circles in the scatter plots indicate MCV that decreased by ≥ 1 SD, and blue circles indicate SCV that decreased by ≥ 1 SD

MCV was positively correlated with the fingertip dexterity test scores (*rs* = 0.630, *p* < 0.001), but showed no correlation with absolute error (*rs* = − 0.051, *p* = 0.797), subjective complaints of pain (*rs* = 0.010, *p* = 0.959), motor function (*rs* = − 0.244, *p* = 0.210), and numbness (*rs* = 0.335, *p* = 0.081). SCV showed a negative correlation with subjective complaints of pain (*rs* = − 0.398, *p* = 0.036) and motor function (*rs* = − 0.515, *p* = 0.005), but not numbness (*rs* = 0.010, *p* = 0.959) (Fig. 5). SCV neither corelated with absolute error (*rs* = 0.047, *p* = 0.814) nor fingertip dexterity test scores (*rs* = 0.167, *p* = 0.394) (Fig. 5).

**Fig. 6:**
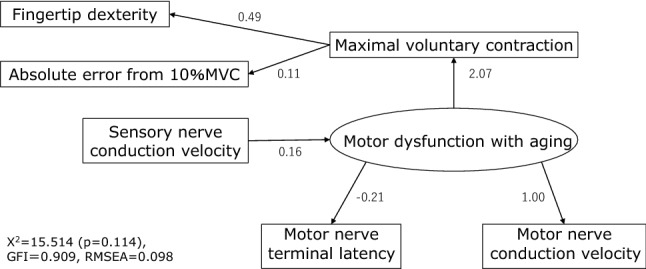
Estimated model using structural equation modeling. The analytical model included the direct effects from maximal voluntary contraction (MVC) on absolute error and fingertip test scores and the indirect effects from motor nerve conduction velocity, motor nerve terminal latency, and sensory nerve conduction velocity on MVC via the assumed aging-associated motor dysfunction. The model generated a *p* value of 0.114, fitting the χ^2^ value (15.514), with a goodness of fit index (GFI) of 0.909 and root mean square error of approximation (RMSEA) of 0.098, confirming model fit. Latent variables are shown as ovals, observed variables as rectangles, and causal relationships as arrows. The numbers adjacent to the arrows represent the strength of the relationship between the estimated variables, and the higher the number (absolute value), the stronger the relationship. "Motor nerve conduction velocity" has a positive effect on “Motor dysfunction with aging”. Therefore, we interpreted a decrease in “Motor nerve conduction velocity” as being related to “Motor dysfunction with aging”. On the other hand, “Motor nerve terminal latency” has a negative effect on “Motor dysfunction with aging” despite the fact that it is an index with no negative values, so it is considered unlikely to be involved in "Motor dysfunction with aging. “Sensory nerve conduction velocity” indirectly affects “Maximal voluntary contraction”, but to a lesser extent than having direct affect from “Motor dysfunction with aging”. In other words, “Motor dysfunction with aging” had a stronger effect on the decrease in “Maximal voluntary contraction. Finally, “Maximal voluntary contraction” was positively affect with “Finger dexterity” and “Absolute error from 10% MVC, and we interpreted the decrease in “Maximal voluntary contraction” as being more likely to affect the decrease in “Finger dexterity”

An analytical model was constructed using the six variables (MCV, SCV, motor nerve terminal latency, MVC, absolute error, and fingertip dexterity test scores) and one latent variable (motor nerve impairment) that showed differences between the young and elderly groups, and then structural equation modeling was performed. Structural equation modeling is a hypothesis-driven method generating an estimated model, which is described here and shown in Fig. 6. Although many of the relevant prior studies are described below and were in a fragmented, experiment-based setting, each study was consistent and a single hypothetical model was assumed by piecing together the prior studies. In order to understand the validity of the hypothetical model derived from the prior studies, it was important to examine the relationships among the multiple factors involved. The insufficient activation of motor units, i.e., inadequate rate at which motor units generate action potentials, is generally observed with aging, and this may contribute to the age-related decline in muscle strength and power (Hunter et al. 2016), which we considered was associated with “Motor dysfunction with aging” to “Motor nerve terminal latency” and “Motor nerve conduction velocity.” However, motor nerve terminal latency is the combined final time of the conduction, transmission, and evocation of action potentials, and is dependent on the length of the participant's hands. Therefore, it was hypothesized that, unlike MCV, it is not related to finger dexterity. In addition, motor nerve conduction velocity was never implicated in reduced fingertip dexterity, ignoring reduced pinch force, we did not assume a causal relationship between these factors and fingertip dexterity (Marmon et al. 2011). Sensory nerve impairment affects the feedback control of movement (Kelso 1982), which we considered was associated with “Sensory nerve conduction velocity” to “Motor dysfunction with aging” The reduction in muscle strength associated with decreased muscle output increases the unsteadiness of muscle output during movement (Enoka et al. 2003), and muscle weakness causes a reduction in fingertip dexterity (Marmon et al. 2011), which we considered was associated with “Motor dysfunction with aging” to “Fingertip dexterity” and “Absolute error from 10% MVC” via "Maximal voluntary contraction." The analytical model included the direct effects of MVC on absolute error and fingertip test scores and the indirect effects of MCV, motor nerve terminal latency, and SCV on MVC via the assumed aging-associated motor dysfunction. The model generated a *p* value of 0.114, fitting the χ^2^ value (15.514), with a goodness of fit index of 0.909 and root mean square error of approximation of 0.098, confirming model fit.

## Discussion

### Results and interpretation of this study

The elderly participants in this study did not have clinically diagnosed disease, and it was expected that MCV and SCV would not change significantly with age. However, minor decreases in MCV and SCV were observed, albeit within the normal range of age-related changes. Normal age-related pathological changes in peripheral nerves reduce the overall number of axons and neurons, with a decrease in conduction velocity, but not less than 70% of the lower limit of normal (Kimura 2013), and a decrease in velocity of only approximately 10 m/s, corresponding to a reduction in the number of myelinated nerves due to aging (Kimura 2013). Mayer (1963) reported that the MCV (mean ± SD) of the median nerve at the elbow to wrist was 59.3 ± 3.5 m/s for young individuals and 54.4 ± 4.0 m/s for elderly subjects, and the SCV of the median nerve was 67.7 ± 4.4 m/s and 62.8 ± 5.4 m/s, respectively. In a somewhat more recent study (Chang et al. 2004), the MCV and motor nerve latency (from the palm) of the median nerve at the elbow to wrist of healthy participants, set as a control group, were 57.82 ± 3.9 m/s and 3.66 ± 0.3 ms, respectively, although the age range of the target group was wider than that of Mayer (1963). The corresponding results of the present study for the young and elderly groups were an MCV (mean ± SD) and motor nerve latency (from the palm) of the median nerve at the elbow to wrist of 55.8 ± 3.7 m/s and 52.2 ± 5.0 m/s and 2.16 ± 0.43 and 2.57 ± 0.75 ms, respectively, with a median SCV of 59.4 ± 3.4 m/s and 55.5 ± 5.3 m/s, respectively, which are generally similar to those previous studies. Furthermore, even when the focus is on differences between young and elderly adults, the MCV and SCV were not below 70% of the lower limit of normal, and in addition, they remained within 10 m/s. Thus, in the present study, we observed a significant decrease in MCV and SCV, but the decrease in conduction velocity was minor, and appears to be due to normal aging. However, one notable finding is that subjective complaints of pain and motor function were correlated with decreased SCV. In addition, the elderly participants had decreased MCV and delayed motor nerve terminal latency, and the decrease of MCV correlated with decreased fingertip dexterity. The elderly group was characterized by larger absolute errors and lower fingertip dexterity test scores, and the reduction of fingertip dexterity was attributed to a lower pinch force, which was presumed to be causally related to neurophysiological influences in sensory-motor coupling.

### Subjective complaints and fingertip dexterity in elderly individuals

Even in the absence of a medical diagnosis, a decrease in fingertip dexterity significantly reduces the ADLs and QOL (Canning et al. 2000; Duque et al. 1995). The participants in this study were healthy elderly individuals without a medical diagnosis, but they had subjective complaints of pain, motor function, and numbness. Furthermore, compared to the young group, the elderly group showed increased absolute error during the pinch force regulation task at 10% MVC and decreased fingertip dexterity test scores on the pegboard, indicating lower fingertip dexterity. However, since no age-related differences were observed in the integral values of the sweating waveforms, it is difficult to believe that the decrease in fingertip dexterity was a transient reduction due to mental strain (Michimata et al. 2008). In general, elderly subjects show a marked decline in fine motor skills rather than in gross motor skills (Smith et al. 1999), and in a task of manipulating cylinders of different sizes, elderly participants have difficulty in grasping and operating cylinders as smoothly as young participants do (Bennett and Castiello 1994). Muscle weakness reportedly induces such a decline in fingertip dexterity (Marmon et al. 2011). Muscle strength decreases with age (Lexell 1997; Mathiowetz et al. 1985), and muscle weakness is associated with a decrease in force coordination ability and fingertip dexterity as assessed by a pegboard task (Hamilton and Wolpert 2002; Marmon et al. 2011). In the present study, the elderly participants also showed a decrease in MVC in pinch movements (Lexell 1997; Marmon et al. 2011; Mathiowetz et al. 1985), and it was inferred that this muscle weakness affected their ability to adjust pinch force, as well as their ability to grasp and manipulate objects, leading to a decrease in the ADLs and QOL, which was reflected in their subjective complaints. However, the results of this study differed from those of previous studies. Marmon et al. (2011) reported absolute error as being related to fingertip dexterity test scores, but we found no such correlation regardless of age. This is because the pegboard used to calculate fingertip dexterity test scores in the present study was relatively larger than those used in the previous study, and it was necessary to use not only the fingertips but also the entire upper limbs in a comprehensive manner, so the ability to adjust force as an index of absolute error was only one factor in the pegboard task (Aaron and Jansen 1992). However, in any case, it was clear that many elderly individuals, even those without a medical diagnosis, have subjective complaints of pain, motor function, and numbness as they age, and that fingertip dexterity also actually declines.

### Relationship between nerve conduction velocity, subjective complaints, and fingertip dexterity

Mayer (1963) reported an MCV (mean ± SD) of 54.4 ± 4.0 m/s and SCV of 62.8 ± 5.4 m/s in the forearms of 51–80-year-old subjects, and considering these as normal values for the elderly, the nerve conduction velocity in this study was not abnormal (decrease of ≥ 2 SD). Several elderly participants were at the lower limit of normal (1–2 SD). Compared to the young participants, the elderly subjects had larger absolute errors and lower scores in the fingertip dexterity test, so-called manual dexterity. In particular, many of those with low fingertip dexterity test scores also had an MCV at the lower limit of normal, which was thought to be related to the positive correlation between MCV and fingertip dexterity test scores; the decrease in SCV, another common aging-associated change, was also above the lower limit of normal. Motor nerves do not propagate sensory information and are not likely to be associated with sensory aspects such as pain and numbness. However, since motor nerve abnormalities may induce a loss of synchrony between the activity of multiple muscles, we considered that the reduction of motor skill was associated with the fingertip dexterity test score for complex movements rather than the absolute error for simple pinch movements. There was no correlation between the decrease in SCV and the decrease in manual dexterity, but there was a negative correlation between subjective complaints, especially pain, and motor function. Regarding pain, normal aging-associated changes in sensory nerves may be influential. Conversely, regarding motor function, although there seems to be no relationship between sensory nerves and motor function, sensory nerve disorders affect the feedback control of movement and thus adversely affect motor performance (Kelso 1982). Afferent information from peripheral sensors is combined with predictive signals generated by an internal motor model (Wolpert et al. 1995). Therefore, changes in the accuracy of sensory information relative to predicted movement outcomes will have widespread effects on motor control (Edwards et al. 2012). Normal aging results in reduced sensory sensitivity and increased sensory noise in peripheral and central processing (Konczak et al. 2012). Aging decreases somatosensory sensitivity, especially during self-generated movement (Klever et al. 2019; Wolpe et al. 2016). The relatively large increase in predictive signal accuracy with age may be an adaptive mechanism of healthy aging (Moran et al. 2014). As sensory variability increases and experience accumulates with age, elderly participants may rely more on internal predictive models (Klever et al. 2019). Unless offset by greater weighting of the internal model, the noisy sensory information associated with aging may otherwise lead to a reduced ability to distinguish between self- and externally-induced sensations, resulting in the abnormal attribution of behavior (Klever et al. 2019). The failure to investigate the intensity of sensory input directly using a robust quantitative measure of sensory-motor attenuation (e.g., force-matching task (Wolpe et al. 2016), the processing of externally generated stimuli on a moving limb (Fuehrer et al. 2022)), and tactile suppression (Klever et al. 2019) is a weakness of this study. Although, the sensory nerve disorders in the elderly participants would imply an age-related modulation of the underlying sensory afferents, and it has been related to subjective functional complaints because they required unreliable estimations and excessive movement adaptations, even if they did not reduce absolute error or fingertip dexterity test scores, which are actual measures of motor skills. However, decreased SCV was not associated with numbness, which can be caused not only by decreased conduction velocity due to peripheral neuropathy but also by increased nerve excitability and spontaneous firing of sensory nerves (Yamashita and Ando 2013). In other words, numbness is described using terms such as "tingling" and "zinging," but the types of numbness are very diverse, and it is likely that there is a mixture of participants who describe hypoesthesia as numbness and those who describe sensory sensitivity as numbness. There is reportedly no relationship between subjective symptoms of numbness and the degree of neuropathy (Franse et al. 2000), and it was inferred that numbness was not associated with decreased SCV in the present study.

According to these observations, by interpreting the estimated model generated by structural equation modeling (Fig. 6), a decrease in nerve conduction velocity leads to a decrease in the MVC of pinch movements, resulting in decreased muscle strength, which leads to unstable muscle output and increased variability in muscle output during movements, resulting in higher absolute error and lower fingertip dexterity test scores. However, the model also showed that motor nerve terminal latency did not play a significant role in this process. Three components are involved in motor nerve terminal latency: (1) conduction time from the stimulation site to the nerve terminal; (2) time for transmission to the muscle endplate at the neuromuscular junction; and (3) depolarization of the endplate followed by the evocation of action potentials at the muscle cell membrane. Conversely, sensory nerve terminal latency reflects only the conduction time from the stimulation site to the rise of the negative waveform. In the present study, despite the fact that MCV and SCV were similarly decreased in the forearm segment, terminal latency was only delayed in motor nerves, which may be the result of the influence of factors (2) or (3), which are not common to both. However, SCV was reduced in the forearm, which does not suggest that the distal portion of the sensory nerve is normal, and terminal latency may be less likely to reflect neuropathy. Conversely, motor nerves had decreased terminal latencies, suggesting that they may be more strongly impaired. Factors other than reduced nerve conduction velocities, including the relationship between increasing age and decreased fingertip dexterity, are caused by the reduced strength and mass of skeletal muscles (Brown et al. 1995; Carmeli et al. 2003; Enoka et al. 2003; Grabiner and Enoka 1995; Martin et al. 2015), and factors on the skeletal muscle side that delay action potential generation include decreased resting membrane potential and increased threshold and decreased activity and efficiency of Na^+^–K^+^ pumps (De Luca et al. 1990; Frolkis et al. 1976; Kjeldsen 1987). In the present study, in which no continuous high-frequency electrical stimulation was given, component (2) was unlikely, and it was inferred that the influence of component (3) was significant. Thus, assuming that the main component of the delayed terminal latency is the time required for action potentials to be evoked at the muscle cell membrane following endplate depolarization (3), the estimated model does not significantly interfere with the decrease in fingertip dexterity, suggesting that a decrease in nerve conduction velocity at or above the lower normal limit is still an important indicator of decreased fingertip dexterity. One weakness of this study, however, is that the medical diagnosis of disease in the elderly was limited to interviews. The intent was to prove that potential problems exist even in elderly participants who are only consciously healthy. However, elderly participants are normally on medications for hypertension, diabetes, depression, etc. Therefore, a limitation is that pharmacological factors were likely included in the results of this study.

## Conclusion

Decreased fingertip dexterity may be related to decreased pinch force due to neurophysiological factors. Even in elderly subjects without a medical diagnosis, the conduction velocity of motor/sensory nerves was slightly reduced. While this was within the general category of age-related changes, occasional decreases of conduction velocity at or above the lower limit of normal may be linked to decreased fingertip dexterity and the presence of subjective complaints and should be noted. Potentially, complaints of sensory abnormalities and finger dexterity could be resolved by identifying a minor decrease in conduction velocity. Furthermore, it would be a significant finding to confirm age-related abnormalities in fingertip dexterity, including subjective complaints, from conduction velocity at the forearm segment, where stable measurements can be made.

## Data Availability

The datasets generated during and/or analyses during the current study are available from the corresponding author on reasonable request.
